# Anxiety and Metabolic Disorders: The Role of Botanicals

**DOI:** 10.3390/cimb45020068

**Published:** 2023-01-28

**Authors:** Hanna Trebesova, Valentina Orlandi, Raffaella Boggia, Massimo Grilli

**Affiliations:** 1 Department of Pharmacy, University of Genoa, Viale Cembrano 4, 16148 Genoa, Italy; 2 Inter-University Center for the Promotion of the 3Rs Principles in Teaching & Research (Centro 3R), 16148 Genoa, Italy

**Keywords:** anxiety, metabolic disorders, nutraceuticals, botanicals, bud derivates

## Abstract

Anxiety and anxiety-related disorders are becoming more evident every day, affecting an increasing number of people around the world. Metabolic disorders are often associated with anxiety. Furthermore, anxiety branches into metabolic disorders by playing multiple roles as a cofactor, symptom, and comorbidity. Taken together, these considerations open the possibility of integrating the therapy of metabolic disorders with specific drugs for anxiety control. However, anxiolytic compounds often cause disabling effects in patients. The main goal could be to combine therapeutic protocols with compounds capable of reducing side effects while performing multiple beneficial effects. In this article we propose a group of bioactive ingredients called botanicals as a healthy supplement for the treatment of metabolic disorders related to anxiety.

## 1. Introduction

According to the World Health Organization, in 2019, 301 million people worldwide suffered from an anxiety disorder. Anxiety affects 4.6 percent of women and 2.6 percent of men [1]. The recent COVID-19 pandemic has exacerbated the problem with social, age and gender implications. Anxiety often affects young people, but the decline in incidence among older people is probably the result of a large underestimatation.

Nevertheless, the most representative model is seeing adults less affected. The complexity of anxiety disorder treatment is also due to the high comorbidity between anxiety and depressive disorders [2]. Therefore, anxiety disorders frequently remain underdiagnosed and under-treated. Anxiety can currently play three important roles affecting human health: a disease, symptom, and contributing factor. The intricacy of its involvement in the widespread disease becomes particularly clear when we consider the link with metabolic disorders. Metabolic disorders are disarrangements of physiological metabolism, including changes in glucose metabolism, obesity, increased blood pressure, dyslipidemia, insulin resistance, hyperglycemia, and hyperuricemia. As if this was not enough, the drug treatment of anxiety has been gradually revised concerning the elevated risk of side effects. Current guidelines do not recommend benzodiazepines as first-line therapy. Indeed, selective serotonin or serotonin–norepinephrine reuptake inhibitors are proposed. The effects of benzodiazepines on cognition, motor coordination and general health are well-documented in the literature, particularly regarding acute effects. In terms of cognitive deficits, benzodiazepines can produce episodes of amnesia and memory impairment. The need for new therapeutic strategies that address all the pathological roles of anxiety, reducing the possibility of side effects, is evident. In this paper, we analyze the possibility of using active components of plants, also called “botanicals,” to counteract anxiety-related symptoms in metabolic disorders and synergically mitigate metabolic disorders themselves [3]. Even if the term “botanical” is not univocally defined, according to the European Food Safety Authority (EFSA) [4] it includes whole, fragmented or cut plants, plant parts, fungi and lichens or their extracts, whose active molecules’ concentrations can vary greatly. These biologically active compounds, also referred to as nutraceuticals, can be used as ingredients in medicinal products, but also in food products with mutually exclusive regulation in the European Union [5]. In the food sector, nutraceuticals are present both in functional foods and in dietary supplements [3].

## 2. Supporting Data 

**Figure 1:**
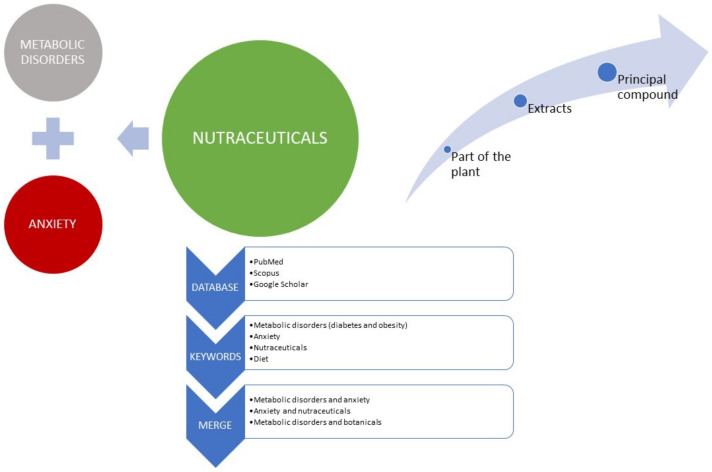
Perspective flowchart.

The perspective is supported by literature that was gathered using the PubMed, Scopus, and Google Scholar databases, as well as from papers cited within the initial articles retrieved. Search terms included combinations of the following words: Metabolic disorders, Anxiety, Metabolic disorders and Anxiety, Metabolic disorders and nutraceuticals, Anxiety and nutraceuticals, Diet, microbiota, and botanicals (Figure 1). 

The impact of metabolic syndrome (MetS) across geographic regions was recently determined by Noubiab and co-workers [6]. Their meta-analysis considered four inclusion criteria ((1) cross-sectional studies; (2) crude prevalence of MetS; (3) adult patients (18 years or more); (4) at least 50 participants) and six regional distributions (Africa Region, Region of the Americas, South-East Asia Region, European Region, Eastern Mediterranean Region, and Western Pacific Region). Data from 1129 prevalence datasets representing 28 million participants show that MetS and individual disorders are very common worldwide. However, the significant variation in the geographic distribution of these disorders suggests the presence of population or country-specific factors, including genetic and cultural factors. With this perspective, we analyzed phytocomplexes and secondary metabolites (Section 4) and isolated active compounds (Section 5).

## 3. Metabolic Disorders and Anxiety

The consequences of metabolic disorders affect individuals, families, and communities, but also health systems and budgets. They affect both industrialized or developing countries. As already mentioned, they include a wide variety of pathologies such as diabetes, obesity, hypertension, hypercholesterolemia, etc., and the causes can be multiple, but diet is an essential factor [7,8]. Interestingly, metabolic disorders may be associated with anxiety and play a role in comorbidity [9]. For example, Type 2 diabetes and obesity could exacerbate psychiatric diseases [10]. To understand the impact of these combinations, it is important to highlight that the World Health Organization estimates that 422 million adults had diabetes in 2019 (WHO) [11]. At the same time, data on people suffering from anxiety disorders and or obesity are growing.

It is well-documented that a poor diet contributes to obesity and increases the risk of anxiety, depression, and mood disorders [12]. In 2018 Sharma and colleagues observed that a 3 week switch to a high-fat diet caused poor physiological outcomes such as weight gain and worsening of blood parameters in rats. The animals showed increased anxious and depression-like behavior as assessed by Open Field and Elevated Plus Maze tests [13]. From a biochemical perspective, the researchers documented an increase in brain fatty acids and a decrease in brain-derived neurotrophic factor (BDNF) and Neuropeptide Y (NPY). Both biomarkers are positively modulated by docosahexaenoic acid (DHA), and dietary supplementation was able to enhance their expression [14,15].

The link between nutrition and the development of disorders is remarkable. Diet and gender have been reported to promote different bacterial populations in the gut [16]. The composition of the microbiota is even involved in the pathogenesis of brain disorders such as depression and anxiety [17]. For this reason, the gut plays a crucial role in the interplay between metabolic disorders and anxiety. It has been proposed that the enteric microbiota may be responsible for insulin resistance in Type 2 diabetes and inflammatory processes [16]. A plant-based diet and low glucose intake lead to weight loss, which is fundamental to this type of disease [12]. The increase of beneficial bacteria with supplementation with probiotic and/or prebiotic ingredients can work synergistically on different comorbidities [18]. A proper diet appears to prevent damage to the intestinal epithelium, as well as prevents metabolism dysregulation [8]. In this context, we should consider genetic predispositions and hormonal dysregulation as key factors in the dietary strategy [19]. Recently, Zu and collaborators underlined a prominent connection between Type 2 diabetes and Alzheimer’s disease [20]. In this context, it is well-documented that anxiety in Alzheimer’s patients can occur in the early stages of the disease [21]. As far as anti-obesity therapy, drugs acting on the CNS affect catecholaminergic and serotonergic transmission. These drugs produce serious side effects, especially when taken along with antidepressants such as selective serotonin reuptake inhibitors or monoamine oxidase inhibitors. In the worst cases, coadministration causes psychiatric disorders and cognitive impairment [22]. In 2021 Zhong and colleagues’ study linked the development of metabolic disorders in depressed subjects and their comorbidity with anxiety. This study confirmed that aging, pre-existent psychiatric symptoms, and changes in endocrine hormones are related factors in the metabolic syndrome affecting anxiety patients. The interplay between all these conditions exacerbated the severity of these patients’ symptoms, which could result in suicide attempts [23,24].

## 4. Botanicals

Botanicals can be naturally present or industrially added ingredients in functional foods, whose consumption as an integral part of the normal diet, and can provide further beneficial health effects on the functionality of human organs or systems, with respect to traditional nutrition sources. Botanicals can also be included in concentrated forms in food (or dietary in the US) supplements (marketed in “dose” form, e.g., pills, tablets, capsules, liquids in measured doses) to supplement the normal diet by increasing the total intake of healthy substances, providing positive health effects without showing therapeutic activity or significantly modifying physiological functions [25]. Food supplements may contain a single plant ingredient or a mixture of them.

Bioactive compounds can be found in fruits, seeds, leaves, buds, oil etc., and they are often referred to as nutraceuticals, a broad umbrella term, usually used to describe any compound derived from food sources with extra health benefits in addition to the basic nutritional value found in foods [26]. Some of these bioactive compounds can also be used as ingredients in medicinal products, but with mutually exclusive regulation with respect to food sector in the European Union [5,27].

Different natural sources are characterized by alternative pools of compounds. Interestingly, various active substances extracted from plants have proven anti-obesity activity, such as resveratrol, epigallocatechin-3-gallate (ester of gallic acid), quercetin, ellagic acid and anthocyanins. Konstantinidi and Koutelidakis reported several animal studies using these compounds for body weight control and obesity reduction. Moreover, the authors evaluated the clinical studies in terms of the effects of coffee and caffeine on body weight control and obesity in humans [28]. Natural compounds are also used to potentially modulate several chronic disorders such as hypertension and hypercholesterolemia. For this purpose, red yeast rice (RYR) is used to aid in lowering cholesterol levels through a statin-like mechanism [29]. In this context, bioactive compounds can contribute to lowering lipid intake and activity, improving endothelial conditions, and reducing cholesterol [30]. However, it is important to highlight that the lactonic form of monacolin K, which is the main bioactive of RYR, has the same chemical structure as the drug named lovastatin. Thus, the EFSA revised it and concluded that the intake of 10 mg of monacolin K with a food supplement would lead to the intake of a therapeutic dose with the same risk of adverse events, involving the musculoskeletal system and liver, as statins. For this reason, to date, in the UE, the levels of monacolin K allowed in RYR-based supplements have been limited (3 mg/day) [31].

Often, plants or part of plants taken as dietary supplementation are bio-transformed into their active metabolites by the gut microbiota [32]. This is the case for ellagic acid, which is metabolized into urolithins that seem to mediate its multiple therapeutic effects [33].

Among the botanicals, bud extracts have an emerging role in the market, but they are still poorly investigated [34,35,36]. The meristematic tissues of plants are enriched from strategic compounds that are often distinct from those found in mature leaves. Due to the lack of information, several scientists have adopted HPLC analysis and UV-Visible fingerprints to distinguish the composition of the bud extract and confirm the botanical source [37,38].

### 4.1. Curcuma Longa

Curcumin, a major diarylheptanoid and polyphenolic component of *Curcuma longa* (Table 1), possesses several pharmacological properties, including antidiabetic and anti-atherosclerotic actions [39,40]. B. Lee and H. Lee reported that curcumin was able to improve animal scores in the Open Field and Elevated Plus Maze tests. From a mechanistic point of view, the diarylheptanoid restored the 5-HT levels in the hippocampus, amygdala, and striatum. These results suggest that curcumin possesses anxiolytic-like effects on biochemical and behavioral symptoms related to anxiety [41]. On the other hand, curcumin can reduce fatty acid oxidation, adipocyte apoptosis, and AMPK activation. Moreover, it can decrease the expression of peroxisome proliferator-activated receptors-g (PPARg) and CCAAT/enhancer-binding protein a (C/EBPa). Finally, it inhibits pancreatic lipase activity and has improved metabolic conditions in obesity and glycemic control in Type 2 diabetes in mouse models [40,42,43]. In case of impaired liver function, or biliary or biliary tract stones, the use of the product is not recommended, nor during pregnancy and breastfeeding.

### 4.2. Pimpinella Anisum

From a nutraceutical perspective, another interesting plant is *Pimpinella anisum* (Table 1). Based on a long tradition of use, the plant is considered safe [44]. In their article, Es-Safi and coworkers discovered that a total extract of *Pimpinella anisum*, containing different compounds, such as gallic acid, catechin, caffeic acid and quercetin, exhibits antidepressant and anxiolytic effects without impairing memory. This study suggests monoaminergic modulation as a possible mechanism of action [45]. Meanwhile, in the diabetic rat model, anise extracts have considerably decreased body weight and ameliorated blood glucose and serum amylase, acting as a hypoglycemic and antioxidant, with the latter property preserving cells from apoptosis [46].

### 4.3. Trigonella Foenum-Graecum

One interesting finding concerns Fenugreek (*Trigonella foenum-graecum,* Table 1) seeds, which have been reported to have hypoglycemic and cholesterol-lowering effects. Knott and colleagues showed that fenugreek seeds improve the high-density/low-density lipoproteins ratio in high fat-fed mice without affecting circulating levels of total cholesterol, triglycerides, or glycerol, and do not alter glucose tolerance in high fat-fed mice. However, no effects on body weight, fat mass or food intake were observed [47]. Interestingly, 4-hydroxyisoleucine from Fenugreek seeds was reported to be effective on depressive- and anxiety-like behaviors in socially isolated rats in a study proposed by Kalshetti [48].

### 4.4. Withania Somnifera

Another traditional plant, used in Ayurvedic medicine, is *Withania somnifera* (Table 1)*,* which is well-known in Indian medicine. It has already been classified as an adaptogen and promotes whole-body homeostasis through a specific pharmacological mechanism [49]. In traditional applications, it has been used for stress and anxiety. The predominant components of the plant that are used are freshly dried roots, but other parts of the plant are also used. Among the active phytochemicals, steroidal lactones are mainly known. The data collected from animal studies were derived primarily from the Forced Swim Test in rodents [50,51]. The administration of root extracts significantly decreased stress levels but also body weight, blood glucose and total cholesterol. Moreover, in humans, the effects were evident in terms of blood pressure and hematologic safety markers, and there was an improvement in sleep quality [52]. The results of using this plant in patients with obesity was confirmed by Choudhary’s group through a double-blind, randomized, placebo-controlled trial. The researchers observed a decrease in stress markers, improved psychological well-being, particularly in overweight individuals, reduced serum cortisol levels and food cravings, and improved eating behavior. Therefore, they recommended the use of this botanical in body weight management for adults under chronic stress and anxiety symptoms [53]. In addition, it was reported that *Withania somnifera* supplementation can be used as an adjuvant in diabetes management and to improve exercise capacity [54]. It is important to note that in some European Countries (i.e., Poland) the following recommendation has been highlighted: “*Withania somnifera* (L.) Dunal root powder can be used in an amount of less than 3 g per day. The maximum content of withanolides cannot exceed 10 mg in the recommended daily portion of the product. An entity placing a given food on the market should attach a quantitative specification confirming the content of the sum of withanolides per the recommended daily portion of the product” [55].

### 4.5. Genus Tilia

Almost 40 species are included in the *Tilia* genus, and the majority are used in traditional medicine for their antispasmodic, sedative, anxiolytic and hypoglycemic activities. *T. cordata* (Table 1) is also used for diabetes treatment. Several species were evaluated from the polyphenolic profile, in particular *Tilia tomentosa* Moench (Table 1) [38]. Recently, we have confirmed that *Tilia tomentosa* bud extracts (TTBEs) have an anxiolytic effect [56]. In this work Hole Board and Light/dark tests were performed to assess anxiety-like behavior in mice. Interestingly, the outcome of the oral supplementation highlighted a gender and age-related response [56]. In a previous study conducted by Allio and colleagues, it was observed that TTBEs activate a chloride current mediated by GABA_A_ receptors and bind to benzodiazepine receptor sites [34]. Interestingly, *Tilia tomentosa* extracts are reported to influence mouse intestine neuromuscular contractility, inhibiting KCl-induced contraction, and suggesting a direct action on the smooth muscle cells. At the same time, the improvement of gut microbiota mitigates mucosal inflammation in bowel disorders. Moreover, Cerantola et al. have hypothesized that *Tilia tomentosa* could modulate the alterations of the small intestinal motor activity by interaction with the cholinergic neurotransmission and modulation of nitric oxide-mediated response [57].

### 4.6. Zingiber Officinale

Ginger, the rhizome of *Zingiber officinale* (Table 1), is used in traditional medicine for the treatment of several diseases [58]. The most important compounds are gingerol and shogaol. In a recent study, the blood–brain barrier permeability of several derivatives was tested and the compounds were detected in the brain after a single oral administration to rats [59]. Moreover, the anxiolytic activity has already been demonstrated [60] and confirmed in rats with spinal nerve ligation [61]. In addition, in this paper a dietary supplementation of ginger root extracts reduced the pain sensitivity after 10 post-operation days. At the same time, the impact on gut microbiota was assessed since the link between the microbiota composition and behavioral alterations has already been demonstrated. A decrease in *Prevotellaceae* UCG-001 was observed, concomitant with less anxiety, previously confirmed by Yang’s study [61,62]. Moreover, ginger supplementation has been investigated in clinical trials in patients with Type 2 diabetes. In the treated groups, a reduction in blood glucose and total cholesterol was registered [63]; additionally, a significant decrease in fasting blood sugar (FBS), glycated hemoglobin (HbA_1_C), systolic blood pressure (SBP) and diastolic blood pressure (DBP) was observed [64,65].

**Table 1:** Summarizes the information for the use of selected botanicals in MetS and anxiety.

Botanicals	Principal Secondary Metabolites Involved in the Anxiolytic-like Effect	Principal Secondary Metabolites Involved in the MetS Effect and in Anxiolytic Effect	Note	Recommendation for MetS Effect	Cautions
*Curcuma longa (radix)* 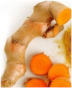		Curcumin	Nano-formulation of curcumin seems crucial to improve its bioavailability. Different effects are mediated by the metabolism induced by different microbiota from person to person	80 mg/day of curcumin in the form of nano-micelles [40]	In case of impaired liver function, biliary or biliary tract stones, the use of the product is not recommended. Do not use during pregnancy and breastfeeding. It is recommended to consult a doctor before its use to avoid possible drug interactions.
	Curcumin as C3 Complex^®^ formula (obtained from Sami Labs Ltd., Bangalore, India) was used as the source of curcuminoids (comprising curcumin, demethoxycurcumin and bisdemethoxycurcumin)			1 g/day for a period of 30 days [66]	In case of impaired liver function, biliary or biliary tract stones, the use of the product is not recommended. Do not use during pregnancy and breastfeeding. It is recommended to consult a doctor before its use to avoid potential interactions with drugs
*Pimpinella anisum (seeds)* 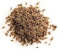		70% hydro-ethanolic extract of previously defatted *P. anisum* powder (mainly containing: *p*-coumaric acid and catechin, followed by oleuropein, gallic acid, caffeic acid, chlorogenic acid, trans-4-hydroxy-3-methoxycinnamic acid, quercetin, and myricetin)		Study on mice model 100 and 200 mg/kg (21 days) [45]	
	Aqueous crude extract			Study on rat model 500 mg/kg orally once daily [46]	
*Trigonella foenum-graecum (seeds)* 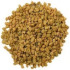		(2S, 3R, 4S)-4-hydroxyisoleucine (4-HI), a major amino acid from fenugreek seeds in saline (0.9% NaCl) solution		Study on rat model 10, 30, 100 mg/kg once a day for 14 days [47]	
				Study on high-fat-fed mice fed a low- or high-fat diet for 16 weeks with or without 2% (*w*/*w*) fenugreek supplementation [48]	
*Withania somnifera (radix)* 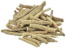		Radix dry extract		300 mg, twice daily (600 mg/day), orally for 8 weeks [49]	
	Radix dry extract (standardized in a complex group of steroidal lactones known as withanolides, which also occur as glycosides withanosides)	commercially available preparations of WS (KSM-66^®^, Sensoril^®^, Essentra^®^, or Shoden^®^)		125–1000 mg daily using capsules for 6–12 weeks	If used as ingredients in dietary supplements in some European Countries (i.e., Poland) the following recommendation has been highlighted “*Withania somnifera (L.) Dunal root powder can be used in an amount less than 3 g per day; The maximum content of withanolides must not exceed 10 mg in the recommended daily portion of the product; An entity placing a given food on the market should include a quantitative specification confirming the total content of withanolides per recommended daily portion of the product* “
*Tilia cordata (flowers)* 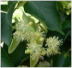				Tilia cordata (1%) [67]	
*Tilia tomentosa Moench (buds)* 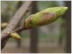	Glyceric Macerate: benzoic acids (47% of the total phytocomplex), catechins (36% of the total phytocomplex) flavonols (14% of the total phytocomplex) and cinnamic acids (3% of the total phytocomplex)			Study on mice Glyceric Macerate dissolved in the drinkingwater (1 µL/mL) for 21 days [56]	
*Zingiber officinale (radix)* 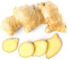		gingerenone A, 6-shogaol, and 6-gingerol [58]			
	ginger root extracts rich in gingerols (GEG) and shogaols (SEG)			Study on rats +0.75%GEG +0.75%SEG in diet for 30 days [61]	

## 5. Plant Bioactive Compounds

### 5.1. Quercetin

Quercetin, already known for its antioxidant, anti-inflammatory, hypoglycemic, and hypolipidemic effects, has shown anti-dementia and neuroprotective effects mainly in rat studies [68,69,70]. Quercetin has been shown to attenuate lipid oxidation, and its anti-inflammatory properties are helpful in obesity and Type 2 diabetes, through the stimulation of glucose uptake while downregulating adipogenesis [71]. Through molecular docking and pharmacological network studies, the researchers have identified several common targets for neurodegenerative diseases and diabetes [20]. Furthermore, quercetin can counteract anxiety as demonstrated in this paper by Samad and colleagues. In their study, in pre-treated mice, quercetin attenuated stress-induced behavior in several tests such as the Light/dark, Elevated Plus Maze, Forced Swimming test and Immobilization stress test. The quercetin administration has also improved mice performance in the Novel Object Recognition test. In addition, the antioxidant enzymes AChE, ACh and 5-HT were evaluated; quercetin could prevent the impairment of antioxidant enzymes, regulate the serotonergic and cholinergic neurotransmission, produce antianxiety, and antidepressant effects and enhance memory [72]. In a recently published work by Chen, the researchers developed a mice model of anxiety-like behavior induced by methamphetamine administration, where quercetin was able to decrease anxiety symptoms and reactive oxygen species (ROS). Chen also demonstrated that quercetin reduces neuroinflammatory effects by decreasing the IL-1β and TNFα levels [73]. However, the EFSA has not currently granted any authorization on the health claim related to the antioxidant activity of foods containing quercetin.

### 5.2. Berberine

Berberine, a natural isoquinoline alkaloid, known in Chinese traditional medicine and present in several plants, is used extensively in Asia for Type 2 diabetes. Recent studies have demonstrated multiple pharmacological activities: hypoglycemia, hypolipidemia, anti-inflammation, and anti-oxidation [74,75,76]. Berberine is often used as an ingredient in association with red yeast to help control cholesterol levels, as reported in the first report sold in Italy [77]. Hsu and colleagues have observed an improvement in insulin resistance by increasing insulin receptor expression, insulin sensitivity and activation of phosphatidylinositol 3-kinase (PI3K)/Akt [78]. However, high glucose levels in diabetic patients can lead to neuronal damage. Thus, the anti-inflammatory and antioxidant properties of berberine may be useful in neurodegenerative diseases. Berberine administration reduced beta-amyloid plaque aggregation in a mouse model of Alzheimer’s disease; some effects have been also reported for Parkinson’s disease. Hsu and colleagues themselves have reported that berberine activates nuclear factor-like 2 (Nrf2), an anti-oxidant factor [79]. The berberine potential has been expanded lately to mood disorders since the involvement of dopamine is documented; nevertheless, the mechanism of action is still unclear. A review by Fan has described berberine’s effects on depression and anxiety. It affects the GABAergic and serotonergic systems [80]. The study on rats with post-traumatic disorder conducted by Lee resulted in an improvement in Elevated Plus Maze and Open Field performance after 14 days of intraperitoneal treatment of berberine extract when subjected to single prolonged stress. From a neurochemical point of view, berberine increases dopamine levels in the hippocampus and striatum. Consequently, the modulation of the dopaminergic system may be an adjuvant treatment in anxiety-like behaviors, even those associated with post-traumatic stress disorder (PTSD) [81].

### 5.3. Caffeic Acid

As a single compound, caffeic acid seems to be a good prospect for either metabolic disorders or anxiety-like behavior. Caffeic acid is a non-flavonoid phenolic compound found in several plants such as coffee, thyme, sage, star anise, seeds and even berries. All sources listed are well-documented in the literature and used in traditional medicine for their antioxidant, immunomodulatory, neuroprotective, anxiolytic, and anti-inflammatory activities, and in turn, they can modulate inflammation and oxidative stress in metabolic diseases [82]. Since the increase in oxidative stress in diabetic patients is associated with a decrease in cellular antioxidant defense mechanisms and hyperglycemia induces an increase in reactive oxygen species (ROS) levels, caffeic acid may be useful in diabetes and other oxidative-stress-related conditions. The counteraction of ROS also plays a crucial role in neuroprotection [83]. Caffeic acid has been implicated in animal models for depression and anxiety studies, showing signaling modulation and inhibiting apoptosis in the hippocampus [84].

### 5.4. Ellagic Acid

The involvement of the GABAergic system in anxiety is well documented. As mentioned before, *Tilia* has shown anxiolytic activity by acting in a benzodiazepine-like manner. Different compounds could be the source of this effect as well as the synergistic action of several substances [85]. Interestingly a flavonoid, ellagic acid (EA), has exhibited similar activity in the Elevated Plus Maze on adult mice after acute and chronic oral treatment [86]. In this paper, the possible mechanism of action was also investigated; ellagic acid showed an anxiolytic effect through the involvement of GABAergic but not the serotonergic system, and the use of different receptor blockers has discerned a possible site of action [86]. Since several diseases are associated with behavioral alteration, sometimes with an anxiety component, Farbood and colleagues have investigated the link between anxiety-like behavior and diabetes in a rat model. Anxiety was evaluated with several behavioral tests, such as the Elevated Plus Maze and Morrison test, and cytokines and neurotrophic factors were assessed. Interestingly, anxiety parameters and locomotor activity were improved in the ellagic group, and only locomotor parameters were affected in insulin-treated rats. EA treatment has helped with glycemic control, which may prevent behavioral and cognitive deficits, and slow hyperglycemic neuroinflammation [87]. The ellagic acid has proved to be effective not only in the diabetic rat model but also in the genetic rat model of metabolic syndrome. In their research, Kabelova and colleagues have observed that rats fed a high-fat diet in combination with intragastric gavage of ellagic acid ameliorated glucose, insulin and cholesterol levels, and oxidative stress levels as well. In addition, in these rats registered a downregulation of the brown adipose tissue markers *Dio2* and *Nr4a1* and an upregulation of insulin-sensitizing gene *Pla2g2a* [88]. For these reasons, ellagic acid is a useful botanical for metabolic syndrome, diabetes and resulting anxiety-like behavior therapeutic support. However, it is important to emphasize that there is a large inter-individual variability in these health effects due to the different types of ellagic acid metabolites that can be produced [89].

## 6. Discussion and Conclusions

Metabolic disorders and anxiety are often two faces of the same coin. Step therapy may involve several drugs with important side effects. Our idea is to introduce supplements in the therapeutic protocols, and in particular botanicals, that are effective in simultaneously modulating aspects of both diseases. A meta-analysis of scientific literature supports this hypothesis, although some considerations are needed: (1) we know that phytochemicals, pre- and probiotics, minerals, and vitamins may contribute to the maintenance of metabolic homeostasis and mental health. (2) The literature presented in the principal scientific databases is still fragmented and we need more information. (3) We must recognize that the information about the safety and effectiveness of these supplements is limited. Indeed, despite compelling animal studies, evidence in humans is restricted (4) It is of utmost importance to define an unequivocal definition of nutraceuticals, mostly referred to as compounds “*beyond the diet but before the drugs*” and shared regulations are urgently mandatory [90]. For this reason, further investigations should be conducted, to define effective dosages, interactions with approved drugs and safety [91]. A recent paper [92] summarizes specific guidelines to produce reliable investigations on the nutraceuticals’ properties of plant derivates. As far as food legislation is concerned, Kusar et al. recently summarized the use of botanicals in functional foods and food supplements [27].

Following these suggestions, we can obtain comparable data supporting the application of nutraceuticals in clinical protocols. However already at present, plant extracts represent the ideal prototype of a multitarget approach [93,94], which is an emerging paradigm in parallel to a specific target strategy. Botanicals are strictly linked to gut microbiota in human health. The relationships between gut microbiota and neurodegenerative, inflammatory and metabolic diseases are being increasingly explored in recent years [95,96,97]. Bidirectional connection is well-documented, demonstrating that microbiota can be modulated by active compounds and, vice versa, the metabolic machinery of bacteria is fundamental in the absorption of natural derivates [98,99]. The literature demonstrates that botanicals can synergize with probiotic supplementation both in a pharmacokinetic and pharmacodynamic manner. Interestingly, in a recent paper, the screening of the *Bifidobacterium* strain done by Duranti and coworkers has revealed that *Bifidobacterium adolescentis* can produce gamma-aminobutyric acid (GABA). This finding underlines a potential therapeutic implication since GABA is a principal inhibitory neurotransmitter in the CNS and plays a key role in anxiety and depression disorders [100]. At the same time, GABA was proposed as a treatment together with sitagliptin to reduce apoptosis of beta cells in diabetic patients [101]. This scenario describes, once more, one possible common denominator between anxiety and metabolic derangements. Many dietary supplements appear to be suitable for the combined treatment of metabolic and anxiety disorders. Basic research and clinical studies must complete the framework to understand the true potential of therapeutic protocols, which increasingly include the support of nutritional experts.

## Data Availability

No new data were created.
